# Genome-Wide Association Study of Lithium-Induced Dry Mouth in Bipolar I Disorder

**DOI:** 10.3390/jpm11121265

**Published:** 2021-12-01

**Authors:** Lawrence Shih-Hsin Wu, Ming-Chyi Huang, Chih-Ken Chen, Chen-Yang Shen, Cathy Shen-Jang Fann, Chun-Yuan Lin, Chih-Chien Lin, Andrew Tai-Ann Cheng

**Affiliations:** 1 Graduate Institute of Biomedical Sciences, China Medical University, Taichung 406040, Taiwan; lshwu@hotmail.com; 2 Taipei City Psychiatric Center, Department of Psychiatry, Taipei City Hospital, Taipei 10341, Taiwan; mingchyihuang@gmail.com; 3 Department of Psychiatry, School of Medicine, College of Medicine, Taipei Medical University, Taipei 110, Taiwan; 4 Community Medicine Research Center, Department of Psychiatry, Chang Gung Memorial Hospital, Keelung 204, Taiwan; kenchen@cgmh.org.tw; 5 College of Medicine, Chang Gung University, Taoyuan 333, Taiwan; 6 Institute of Biomedical Sciences, Academia Sinica, Taipei 115, Taiwan; bmcys@ibms.sinica.edu.tw (C.-Y.S.); csjfann@ibms.sinica.edu.tw (C.S.-J.F.); 7 School of Medicine, Chung Shan Medical University, Taichung 40201, Taiwan; linjyuan@gmail.com; 8 Tsaotun Psychiatric Center, Ministry of Health and Welfare, Nantou 540, Taiwan; 9 Department of Psychiatry, Taichung Veterans General Hospital, Taichung 40705, Taiwan; cclin@vghtc.gov.tw

**Keywords:** bipolar I disorder, lithium, adverse drug reaction, dry mouth, genome-wide association study (GWAS)

## Abstract

Dry mouth is a rather common unpleasant adverse drug reaction (ADR) to lithium treatment in bipolar disorders that often lead to poor adherence or early dropout. The aim of this study was to identify the genetic variants of dry mouth associated with lithium treatment in patients with bipolar I (BPI) disorder. In total, 1242 BPI patients who had ever received lithium treatment were identified by the Taiwan Bipolar Consortium for this study. The proportions of patients who experienced impaired drug compliance during lithium medication were comparable between those only with dry mouth and those with any other ADR (86% and 93%, respectively). Dry mouth appeared to be the most prevalent (47.3%) ADR induced by lithium treatment. From the study patients, 921 were included in a genome-wide association study (GWAS), and replication was conducted in the remaining 321 patients. The SNP rs10135918, located in the immunoglobulin heavy chain locus (IGH), showed the strongest associations in the GWAS (*p* = 2.12 × 10^−37^) and replication groups (*p* = 6.36 × 10^−13^) (dominant model) for dry mouth with a sensitivity of 84.9% in predicting dry mouth induced by lithium. Our results may be translated into clinical recommendation to help identify at-risk individuals for early identification and management of dry mouth, which will improve medication adherence.

## 1. Introduction

Bipolar I (BPI) disorder is a severe, chronic, and disabling mental illness characterized by pathological mood swings with a high tendency to recur [1,2]. Lithium has been placed as a treatment of choice, not only for acute mania and the augmentation of antidepressant in unipolar depression [3], but also for the long-term prevention of manic and depressive recurrences [2,4]. However, the prophylactic benefits from lithium treatment are hindered primarily by its long-term effects on major organ systems, especially the kidneys [5], as well as poor drug adherence [6,7]. Research has shown that non-adherence to lithium is very common, with an average rate of 44.7% (ranging from 18% to 52%) [8,9,10]. One of the commonly reported reasons for the non-adherence to lithium medication is the presence of adverse drug reactions (ADRs) [6,9]. In a five-year prospective study among 402 BPI patients receiving lithium maintenance treatment, 28% of them attributed their premature discontinuation of lithium to ADRs [11].

Although a direct relationship between ADRs and lithium non-adherence still remains unclear because some evidence indicates that patients’ perception of ADRs, as opposed to the actual presence of the ADRs, may contribute more to non-adherence [10] and the tendency of clinicians to attribute non-adherence to ADRs [12]. Nevertheless, nonadherence to lithium prophylactic treatment has been reported to be associated with poorer outcomes in patients with affective-spectrum disorders [13]. As a result, identification of the causes, including genetic predisposition leading to ADRs, as well as careful management and adequate education are imperative to ensure maximal lithium treatment adherence [14]. Given that genetic factors have been shown to play a role in determining the liability to ADRs [15], knowledge of the genetic contribution, when translated into clinical practice, would help palliate their occurrences and maximize the efficacy of drug treatment.

A range of ADRs has been associated with lithium treatment, from less bothersome effects, such as thirst, dry mouth, excessive urination, nausea, diarrhea, and tremor, to severe ADRs, such as weight gain and cognitive impairment, and even serious ADRs, such as renal impairment [16]. Among them, dry mouth and excessive urination are consistently found to be the most common ADRs, with rates up to 70% in long-term treatment [17,18,19,20,21,22]. However, the reasons underlying dry mouth, a condition related to inhibited or decreased salivary flow, are complex. It has been reported that there are inter-individual differences in the ability to perceive the internal bodily state of thirst [23], suggesting that genetic predisposition might also play a role in dry mouth. The exact role of dry mouth in lithium non-adherence and genetic risk factors for this ADR have not been well investigated hitherto.

In this study, we assessed the impact of dry mouth induced by lithium treatment on drug adherence and examined the susceptibility genetic variants for this ADR. We hope that the findings will improve our understanding of lithium-related dry mouth in precision medicine for bipolar disorder.

## 2. Results

### 2.1. Demographic and Clinical Characteristics

Table 1 shows demographic and clinical characteristics of the 1242 study patients, including 580 (46.7%) men and 662 (53.3%) women. The average age of patients was 44.2 (SD = 12.4) years at study entry and 51.9 (SD = 12.7) years at last assessment for ADRs from lithium treatment. Of all patients, 83.0% (*n* = 1031) had experienced impaired drug adherence to lithium and 79.6% (*n* = 821) had at least one type of ADR from lithium. The most frequent ADR was dry mouth (*n* = 588, 47.3%), followed by polydipsia (*n* = 544, 43.8%), tremor (*n* = 323, 26%), gastrointestinal discomforts (*n* = 224, 18%), acne (*n* = 161, 13%), weight gain (*n* = 99, 8%), and hair loss (*n* = 30, 2.4%). The average daily dose of lithium medication was 1145.5 mg (SD: 258.7, range: 800–1800 mg).

### 2.2. Impact of Dry Mouth and Other ADRs on Drug Adherence

As shown in Table 1, among patients with any type of lithium ADRs, 762 (92.8%) had impaired drug adherence during lithium treatment (including dose reduction or discontinuation of lithium). In comparison, only 64% of patients who did not complained of any lithium-induced ADRs had impaired drug adherence (OR = 7.3, 95% CI = 5.2–10.1, *p* < 0.0001). The proportions of patients who experienced impaired drug compliance to lithium treatment were comparable between those only with dry mouth and those with any other ADR(s) (86% and 93%, respectively). As can be expected, significant improvement of ADRs after dose reduction or discontinuation of lithium was observed for dry mouth and any other ADRs (77% and 89%, respectively).

### 2.3. Association Analysis

**Figure 1:**
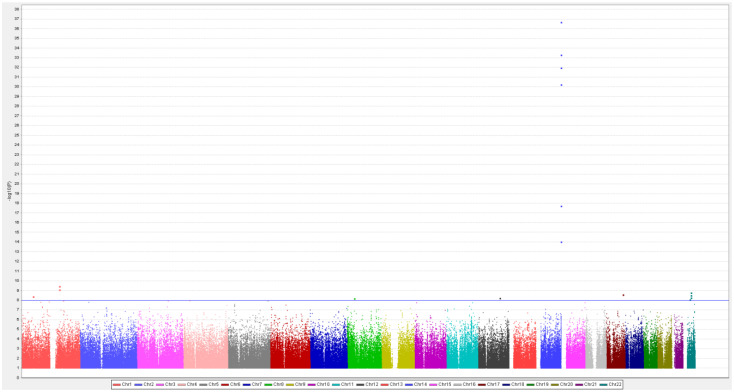
Genome-wide association between single-nucleotide polymorphisms (SNPs) and dry mouth induced by lithium treatment in the GWAS group. The association between individual SNPs and an ADR (dry mouth) induced by lithium treatment in 921 patients with bipolar I disorder is shown. The negative log of the *p* value for the association, as calculated by means of the chi-square test for the dominant model, is plotted against the chromosomal location across the genome. The horizontal line indicates the genome-wide significance level of 1.0 × 10^−8^, which was achieved by several SNPs. The highest SNP on chromosome 14q32.33 is located in the genomic region of immunoglobulin heavy locus (IGH).

The 921 patients who provided genotyping data for GWAS shared a homogeneous genetic background and were unrelated to one another (Figure S1). Eighteen SNPs showed an association with dry mouth induced by lithium treatment with genome-wide significance (Figure 1 and Table S1). The Q-Q plot is shown in Figure S2. The most significant SNP rs10135918 on chromosome 14 is located in the immunoglobulin heavy chain locus (IGH) (*p* = 2.12 × 10^−37^ in the dominant model). The SNPs near rs10135918, including rs7147876, rs10132771, rs7154133, rs2106001, and rs7144717, were clustered in IGH region with association *p*-values ranging from 7.99 × 10^−15^ to 1.41 × 10^−34^. Other SNPs with genome-wide significance ranged from 8.97 × 10^−09^ to 3.83 × 10^−10^ in the dominant model (Table S1).

In the replication group, rs10135918 also showed a strong association (*p* = 6.36 × 10^−13^) (Table 2). We further performed genotyping of rs10135918 in the discovery group to confirm the imputed GWAS genotype data. The *p* value for rs10135918 in the total 1242 study patients was calculated to be 7.41 × 10^−53^ (Table 2).

The predictive values of rs10135918 genotypes for dry mouth induced by lithium treatment are shown in Table 2. Compared to the AA genotype of rs10135918, allele C carriers (CC + AC) were significantly associated with dry mouth from lithium (in the combined group, OR = 7.5, 95% CI = 5.7 − 9.8, *p* = 7.41 × 10^−53^). In all patients, the sensitivity, specificity, positive, and negative predictive values were 84.9, 57.2, 64.6, and 80.8, respectively (Table 2).

## 3. Discussion

In this study, among 1242 BPI patients receiving maintenance treatment of lithium, the most common ADR was found to be dry mouth, which was associated with markedly impaired drug adherence with or without combination medication of other psychotropics. The SNP rs10135918, located in IGH, was found to be strongly associated with dry mouth with acceptable predictive validity. To the best of our knowledge, this is the first study to identify a significant genetic variant for dry mouth induced by lithium maintenance treatment.

Previous work on dry mouth induced by lithium maintenance treatment and its impact on drug compliance has been limited and not analyzed independently from other lithium-induced ADRs, such as thirst, polydipsia, and polyuria. Our study is the first to describe the clinical profile of lithium-treated patients experiencing dry mouth and to focus on its impact on treatment. Of all study patients with impaired drug adherence during lithium maintenance treatment (*n* = 1031), 52.9% (*n* = 545) was associated with dry mouth (Table 1), a figure comparable to previous studies [9,10]. These observations suggest that the impact of dry mouth could result in problems, hindering adherence to lithium therapy, which had been underestimated previously [24]. This study also found a marked improvement in dry mouth after the dose reduction or discontinuation of lithium, consistent with current knowledge that medication adjustment could mitigate ADRs related to lithium [16]. As inadequate drug adherence may lead to an increased risk of relapse, re-hospitalization, greater utilization of healthcare services, and greater mental health care burden [25,26], and poor outcomes including suicide [27], our findings suggest that dry mouth should be cautiously managed in clinical practice.

SNP rs10135918 is located near the immunoglobulin heavy chain variable region 3–48 (IGHV3-48). The human immune system create unlimited antibody specificities to counteract antigens or infectious agents through the somatic rearrangement of gene segments, in particular heavy chains, to form the variable domain region of exons on immunoglobulin genes. The over-representation of IGHV3-48 has been found to be associated with follicular lymphomas [28] and chronic lymphocytic leukemia [29,30] due to its influence in antigen selection with biased expression [28,29]. Elevated frequencies of V_H_ (immunoglobulin heavy chain variable region) rearrangement products were found in different autoimmune diseases, including rheumatoid arthritis, systemic lupus erythematosus, and Sjögren’s syndrome [31]. Among them, Sjögren’s syndrome is a chronic autoimmune disease characterized by exocrine gland dysfunction, specifically the salivary and lacrimal glands, resulting in oral and ocular dryness [32]. Therefore, it is likely that SNP rs10135918, located in the IGHV3-48 gene, influences the immunoglobulin molecular dynamics of B cell programming [33] and is therefore associated with dry mouth. We searched the GTExportal (https://gtexportal.org/home; accessed date 9 November 2021) to reveal the association between genotypes of the most significant SNPs in the IGH region and the expression level of near gene. Although the SNP rs10135918 was not found in the GTEx database, the genotypes of rs7147876 (the second significant variant; Table S1) were found to be associated with the expression level of IGHV3-48. In whole blood, the risk (AA/AG) genotype of rs7147876 has the higher expression level of IGHV3-48 than the GG genotype (https://gtexportal.org/home/snp/rs7147876; accessed date 9 November 2021). Therefore, we speculate that the transcription of IGH V3-48 (or other IGH loci) is affected by these SNPs. Lithium has pro-inflammatory properties, and these immunological characteristics may contribute to the side effects of lithium [34].

This study has some limitations. Although we employed a standardized instrument (the UKU) to assess dry mouth, this instrument is based on patient’s subjective self-report and may not directly associate with objective physiological measurements, such as salivary gland function. Nonetheless, the majority of patients reported marked improvement of dry mouth after dose reduction or discontinuation of lithium. Second, dry mouth induced by lithium probably involves polygenic and environmental factors. In this study, we only present the genetic variant with the strongest association. Overall, the genotypes of rs10135918 show an acceptable predictive power for dry mouth (Table 2).

In conclusion, identification of high-risk patients for dry mouth and other ADRs of lithium followed by adequate psychoeducation with information on these ADRs and their management will enhance drug adherence, treatment efficacy, and outcome. It is recommended that lithium medication for patients with a high risk of ADRs after genetic screening preferably be started from a low dosage with efficient monitoring of blood level and any emerging ADRs for adequate dosage titration.

## 4. Materials and Methods

### 4.1. Study Participants

Study participants came from a total of 1807 unrelated Han Chinese BPI patients, 20 to 65 years of age, recruited from March 2003 to May 2012, from 52 psychiatric departments of general hospitals and psychiatric institutions in the Taiwan Bipolar Consortium (TBC) aimed at the molecular genetic study of BPI and the pharmacogenetic study of mood stabilizers (details have been described previously) [35,36,37]. All patients were referred by their attending psychiatrists and diagnosed according to DSM-IV criteria for BPI disorder with recurrent episodes of mania with or without depressive episode(s). Only patients of Han Chinese descent were considered for the study; ancestry was determined on the basis of oral report by the patients to members of the research team. Patients with other psychotic affective disorders and renal diseases were excluded.

For the pharmacogenetic study of lithium-associated dry mouth, we first identified 1599 patients who had ever received lithium treatment for at least 3 months. Among them, 357 were excluded because they were not suitable for ADR assessment (including loss of contact with patients for further interview assessment on ADRs, no medical chart record of ADRs, persistent concomitant medications or physical conditions during lithium treatment that may have also caused dry mouth, inability to recall or uncertainty about ADRs from patients, refusal from patients to participate in the study, history of renal function impairment, only mild dry mouth was reported, and so forth). The remaining 1242 patients who had good drug adherence throughout the clinical course were included in this study. Among them, 921 patients with genotyping data available were included in the GWAS group and the remaining 321 patients were included in the replication group.

The study was approved by the institutional review board at each of the participating hospitals and at Academia Sinica, Taiwan (IRB approval number: AS-IRB01-050010 and CMUH106-REC2-131). All the study subjects provided written informed consent.

### 4.2. Phenotype Definition and Assessment

Clinical assessment of mania and depression was performed by trained psychiatric nurses and psychiatrists using the cross-culturally reliable and valid Chinese version of the Schedules for Clinical Assessment in Neuropsychiatry (SCAN) [38].

We employed the UKU side effect rating scale [39] to assess ADRs from lithium medication. Dry mouth due to lithium treatment was rated as ‘none’, ‘mild’, or ‘moderate to severe’. The assessment was based on information gathered from a life chart with graphical presentation of lifetime clinical course prepared for every patient recruited by the TBC. This life chart included all manic, hypomanic, and depressive episodes with onset year and month, duration, and severity, all doses of and duration of treatment with psychotropic drugs and mood stabilizers ever prescribed, drug adherence recorded in medical charts during treatment at outpatient clinics, all recorded blood levels of mood stabilizers, history of major physical conditions, and any adverse drug reactions throughout the entire clinical course. This life chart was presented on the basis of integrated information gathered from direct interview with patients and their key family members, interview with in-charge psychiatrists, and a thorough medical chart review.

### 4.3. Genotyping and Imputation

We genotyped DNA samples obtained from the 921 patients in the GWAS group using the Illumina HumanOmni1-Quad BeadChip and the HumanOmni2.5-Quad BeadChip and integrated the two genotype data sets by imputation (for details, see the Supplementary Methods). We genotyped the top SNP from GWAS in the replication group using the Taqman genotyping platform (ABI: Applied Biosystems Inc., Foster City, CA, USA). Reactions were carried out according to the manufacturer’s protocols. The probe fluorescence signals were detected using the ABI Prism 7500 Real-Time PCR System.

### 4.4. Statistical Analyses

Principal component analysis for 921 patients in the discovery group based on the genome-wide IBS (identical by state) pairwise distances was performed using PLINK v. 1.9 (https://www.cog-genomics.org/plink2; accessed date 9 November 2021) [40]. GWAS between patients with and without dry mouth induced by lithium treatment was conducted by chi-square test for the dominant model. The threshold *p* value was set at 1.05 × 10^−8^ after a Bonferroni correction for the number of SNPs (4,750,978). We examined P-value distributions using quantile–quantile (Q-Q) plots. The calculation was performed using PLINK v. 1.9 [40].

## Figures and Tables

**Table 1:** Demographic and clinical characteristics of study patients with bipolar I disorder (N = 1242) *.

Characteristics	Value
Age in years (mean, SD)	
At study entry	44.2 (12.4)
At last assessment (mean, SD)	51.9 (12.7)
Male sex (N, %)	580 (46.2%)
Dry mouth due to lithium medication (N, %)	588 (47.3%)
Impaired drug adherence ** during lithium treatment (N, %)	1031 (83.0%)
Due to lithium ADRs (*n* = 821) (N, %)	762 (92.8%)
In patients with dry mouth and other ADRs (*n* = 362) (N, %)	351 (97%)
In patients with dry mouth only (*n* = 226) (N, %)	194 (86%)
In patients with other ADRs only (*n* = 233) (N, %)	217 (93%)
Without any lithium ADRs (*n* = 421) (N, %)	269 (64%)
Improvement of ADRs after dose reduction or discontinuation of lithium	
In patients with dry mouth and other ADRs (*n* = 351) (N, %)	312 (89%)
In patients with dry mouth only (*n* = 194) (N, %)	149 (77%)
In patients with other ADRs (*n* = 217) (N, %)	193 (89%)

ADR: adverse drug reaction; * only including patients who ever had good drug adherence throughout the clinical course; ** including dose reduction or discontinuation of lithium.

**Table 2:** Predictive validity of rs10135918 genotypes for dry mouth induced by lithium treatment *.

	GWAS Group (N = 921)		Replication Group (N = 321)		Combined Group (N = 1242)	
Genotypes	Dry m (+) ^†^	Dry m (−) ^†^	Dry m (+) ^†^	Dry m (−) ^†^	Dry m (+) ^†^	Dry m (−) ^†^
AC + CC	395	189	104	91	499	280
AA	72	265	17	109	89	374
Total	467	454	121	200	588	654
*p* value for dominant	2.12 × 10^−37^		6.36 × 10^−13^		7.41 × 10^−53^	
Odds ratio (95% CI)	7.7 (5.6–10.52)		7.3 (4.1–13.1)		7.5 (5.7–9.8)	
Sensitivity (95% CI)—%	84.6 (81.0–87.7)		86.0 (78.5–91.6)		84.9 (81.7–87.7)	
Specificity (95% CI)—%	58.4 (53.7–62.9)		54.5 (47.3–61.5)		57.2 (53.3–61.0)	
Positive predictive value (95% CI)—%	67.6 (63.7–71.4)		53.3 (49.1–57.5)		64.6 (61.8–66.2)	
Negative predictive value (95% CI)—%	78.6 (73.9–82.9)		86.5 (80.2–91.0)		80.8 (77.4–83.7)	

* Based on the genotyped data using Taqman genotyping; ^†^ dry m (+): BPI patients with dry mouth induced by lithium treatment; dry m (−): BPI patients without dry mouth induced by lithium treatment.

## Data Availability

The datasets generated during and/or analyzed during the current study are not publicly available due to the limitation of study consent documents with repository deposition but are available from the corresponding author upon reasonable request.

## Supplementary Materials

The following are available online at https://www.mdpi.com/article/10.3390/jpm11121265/s1. Supplementary methods, Table S1: Results of GWAS, list of SNPs with genomewide significance (1.05 × 10^−8^) at dominant model, Figure S1: The principal component analysis plot of the 921 GWAS samples, Figure S2: Q-Q plot of *p* values of the chi-square test from GWAS for dominant model.
