# Supplementary material for: Genome-Wide Association Study of Lithium-Induced Dry Mouth in Bipolar I Disorder
Source: J Pers Med. 2021 Dec 1;11(12):1265. doi: 10.3390/jpm11121265 (PMC8706003; doi:10.3390/jpm11121265)
Supplement: Supplementary file 1 [file jpm-11-01265-s001.zip › jpm-1455690-supplementary.pdf]

Supplementary Materials

Wu LS, Huang MC, Chen CK et al. Genome-wide association study of lithium-associated dry mouth in bipolar I disorder

Supplementary Methods

Genotyping and genotype imputation

Quality control of genotype data

Replication genotyping

Table S1. Results of GWAS, list of SNPs with genomewide significance ($1.05x10^{-8}$) at dominant model

Figure S1. The principal component analysis plot of the 921 GWAS samples

Figure S2. Q-Q plot of p values of the chi-square test from GWAS for dominant model

Supplementary Methods

Genotyping and genotype imputation

Genotyping was performed using the Illumina HumanOmni1-Quad BeadChip (N=936) and the HumanOmni2.5-Quad BeadChip (N=575) by Chun-Tai Co. (Taipei, Taiwan). For this study, we integrated the two genome-wide SNP data sets through imputation with 1000 Genomes. To bridge the two sets, we also genotyped 82 of the first 936 subjects using the HumanOmni2.5-Quad BeadChip. The two gene chips shared about 750K common SNPs. Genotype calling for the two data sets was determined by Beadstudio (Illumina) using default parameters.

The genotype imputation method, IMPUTE2 [1-3], was performed under default setting to estimate the genotypes of SNPs not on array. In the imputation process, reference haplotypes was curated from 1000 Genomes Project Phase III [4]. To improve the efficiency, we perform whole genome imputation in every 5 Mb chunk, respectively. ANNOVAR [5] was used to annotate functional consequences of single nucleotide variants found in our dataset.

Only the data of the 921 qualified subjects according to case selection criteria were used for GWAS.

1. Marchini J. Howie B. Genotype imputation for genome-wide association studies. Nat Rev Genet. 2010;11:499-511.

2. Howie B, Marchini J, Stephens M. Genotype Imputation with Thousands of Genomes. G3: Genes, Genomes, Genetics. 2011;1:457-70.

3. Howie B, Donnelly P, Marchini J. A Flexible and Accurate Genotype Imputation Method for the Next Generation of Genome-Wide Association Studies. PLoS Genet. 2009;5(6):e1000529.

4. Consortium, T.G.P. A global reference for human genetic variation. Nature. 2015;526:68-74.

5. Wang K, Li M, Hakonarson H. ANNOVAR: functional annotation of genetic variants from high-throughput sequencing data. Nucleic Acids Research. 2010;38:e164.

Quality control of genotype data

The following quality control of the genotype data was implemented for each data set to exclude

SNPs and individuals before the imputation.

(1) Individuals with a call rate < 98%;

(2) $P < 1.0 \times 10^{-5}$ for Hardy-Weinberg violation;

(3) SNPs with MAF < 5%;

(4) Samples with first-degree cryptic relationships;

(5) Samples that were potentially contaminated.

Replication genotyping

The dominant model was selected to find the best prediction capacity. Top SNPs from the GWAS of the 921 subjects were further validated using Taqman genotyping (Apply Biosystems, Foster City, CA, USA).

Table S1 GWAS, significant SNPs, Bonferroni correction, list of SNPs with genomewide significance ($1.05x10^{-8}$) at dominant model

SNPs	Nearest gene (s)	feature	Genotype	Count		P value
				Dry mouth	None dry mouth	
rs10135918	IGHV3-48 (IGH locus)	intergenic	AC+CC / AA	395/72	189/265	2.12×10^{-37}
rs7147876	IGHV3-38	intergenic	AA+AG / GG	265/202	80/374	1.41×10^{-34}
rs10132771	IGHV3-49	intergenic	GG+AG / AA	330/137	143/311	1.35×10^{-32}
rs7154133	IGHV3-49	intergenic	TT+CT / CC	333/134	150/304	3.05×10^{-31}
rs2106001	IGHV3-38	intergenic	GG+AG / AA	172/295	54/400	1.46×10^{-18}
rs7144717	ADAM6 (in IGH region)	intergenic	TT+GT / TT	283/184	159/295	7.99×10^{-15}
rs7551957	FCGR2A	intergenic	CC+CT / TT	140/327	59/395	3.83×10^{-10}
rs7511868	FCGR2A	intergenic	AA+AG / GG	141/326	61/393	8.04×10^{-10}
rs5998745	SYN3	intergenic	GG+AG / AA	124/343	50/404	1.71×10^{-09}
rs57176214	RPL38	intergenic	GG+AG /AA	154/313	73/381	2.70×10^{-09}
rs5754442	SYN3	intergenic	AA+AG / GG	124/343	51/403	3.13×10^{-09}
rs137327	SYN3	intergenic	AA+ AC / CC	124/343	51/403	3.13×10^{-09}
rs319986	AGBL4	intronic	CC+CT / TT	83/384	158/296	4.15×10^{-09}
rs4821131	SYN3	intergenic	GG+AG / AA	124/343	52/402	5.65×10^{-09}
rs6538433	SOCS2-AS1	intergenic	CC+CT / TT	83/384	157/297	6.25×10^{-09}
rs4761731	SOCS2-AS1	intergenic	GG+AG / AA	83/384	157/297	6.25×10^{-09}
rs7006983	FAM183CP	intronic	GG+AG / AA	118/349	48/406	6.62×10^{-09}
rs9613515	MN1	intergenic	CC+CT / TT	113/354	45/409	8.97×10^{-09}

Figure S1 The principal component analysis plot of the 921 GWAS samples

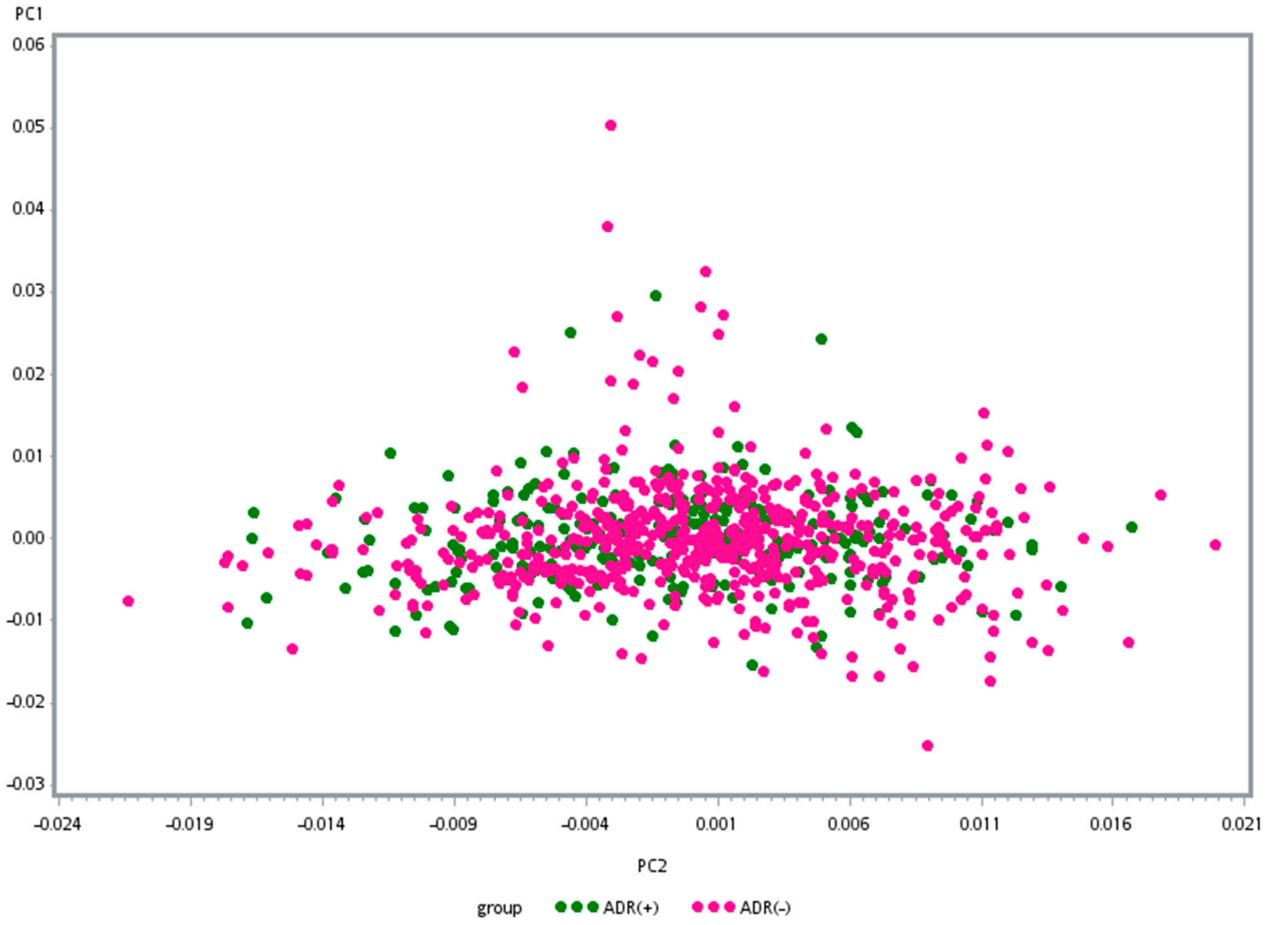

The Y and X axes are the first and second dimensions from principal component analysis (PCA) based on the genome-wide IBS pairwise distances among the 921 GWAS subjects. Pink crosses represented ADR(-) and green for ADR(+).

The two axes correspond to a reduced representation of 10,000 randomly selected SNPs into two dimensions. No clustering pattern was found and indicated that neither substantial population stratification nor cryptic relationship among the 921 subjects was found.

Figure S2 Q-Q plot of p values of the chi-square test from GWAS for dominant model (N=921)

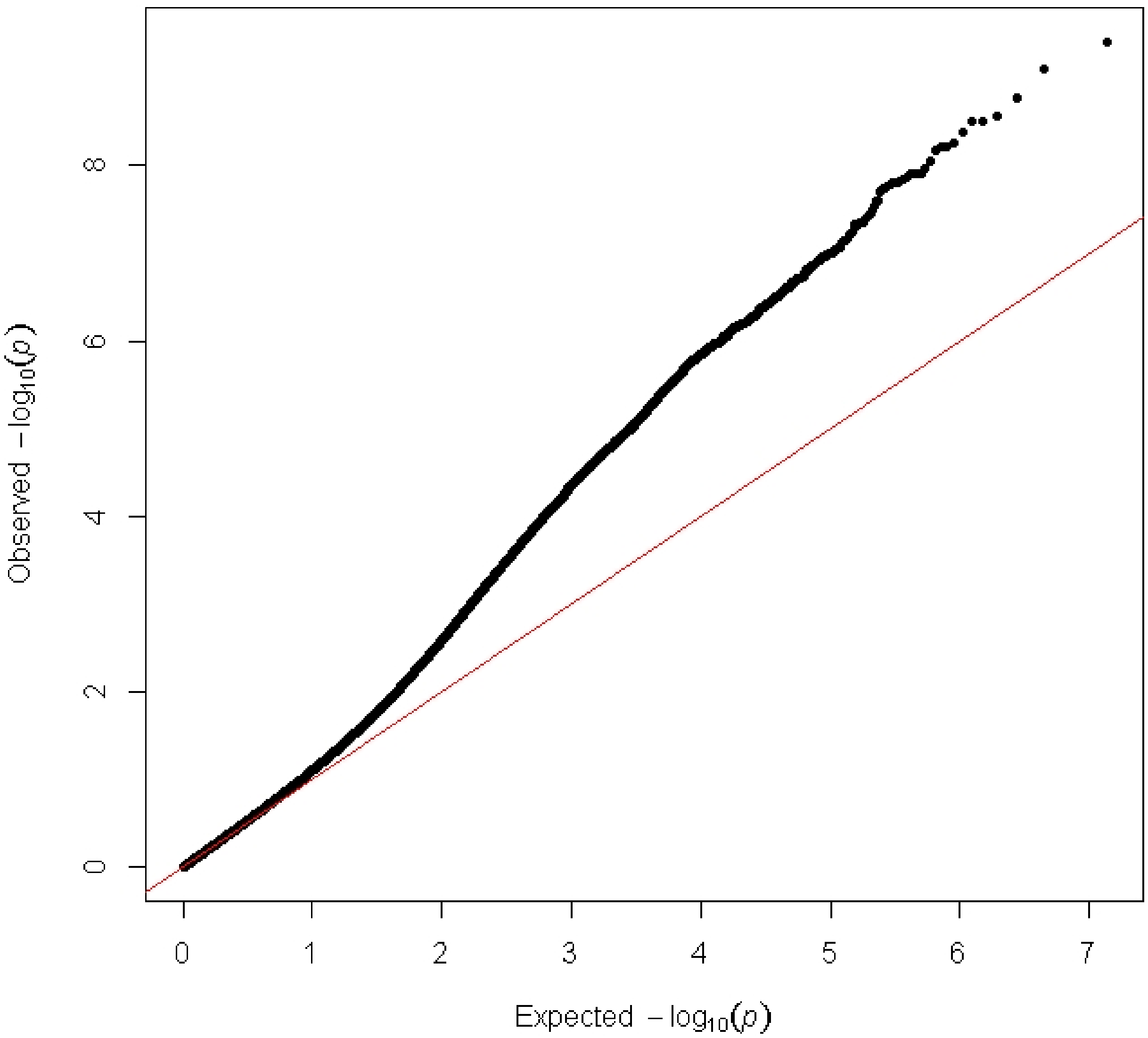

The information of rs10135918 and other associated SNPs in IGH region were not showed in the plot, because its p-value was lower than 10^{-10}.
